# Models and methods: a perspective of the impact of six IMI translational data-centric initiatives for Alzheimer’s disease and other neuropsychiatric disorders

**DOI:** 10.3389/fneur.2023.1174079

**Published:** 2023-07-13

**Authors:** Hilary North, Martin Hofmann-Apitius, Martien J. H. Kas, Hugh Marston, Magali Haas

**Affiliations:** ^1^Scientific Advisor to Cohen Veterans Bioscience, New York, NY, United States; ^2^Department of Bioinformatics, Fraunhofer Institute for Algorithms and Scientific Computing (SCAI), Bonn, Germany; ^3^Groningen Institute for Evolutionary Life Sciences, University of Groningen, Groningen, Netherlands; ^4^Boehringer Ingelheim Pharma, Biberach, Germany; ^5^Cohen Veterans Bioscience, New York, NY, United States

**Keywords:** innovative medicines initiative, pharmaceutical industry, public–private partnership, European Union, neuropsychiatric disorders

## Abstract

The Innovative Medicines Initiative (IMI), was a European public–private partnership (PPP) undertaking intended to improve the drug development process, facilitate biomarker development, accelerate clinical trial timelines, improve success rates, and generally increase the competitiveness of European pharmaceutical sector research. Through the IMI, pharmaceutical research interests and the research agenda of the EU are supported by academic partnership and financed by both the pharmaceutical companies and public funds. Since its inception, the IMI has funded dozens of research partnerships focused on solving the core problems that have consistently obstructed the translation of research into clinical success. In this post-mortem review paper, we focus on six research initiatives that tackled foundational challenges of this nature: Aetionomy, EMIF, EPAD, EQIPD, eTRIKS, and PRISM. Several of these initiatives focused on neurodegenerative diseases; we therefore discuss the state of neurodegenerative research both at the start of the IMI and now, and the contributions that IMI partnerships made to progress in the field. Many of the initiatives we review had goals including, but not limited to, the establishment of translational, data-centric initiatives and the implementation of trans-diagnostic approaches that move beyond the candidate disease approach to assess symptom etiology without bias, challenging the construct of disease diagnosis. We discuss the successes of these initiatives, the challenges faced, and the merits and shortcomings of the IMI approach with participating senior scientists for each. Here, we distill their perspectives on the lessons learned, with an aim to positively impact funding policy and approaches in the future.

## Introduction: overview of the innovative medicines initiative

The IMI Joint Undertaking (IMI-JU) is a European initiative to increase the competitiveness and success of European pharmaceutical research through a unique model that combines intellectual collaboration with academia and public funding.^1^ Officially, the partnership is executed by DG Research and Innovation of the European Commission, of European Communities, and EFPIA (European Federation of Pharmaceutical Industries and Associations). Briefly, the collaborative projects are initiated by participating pharmaceutical companies, who identify a need for collaborative support from the academic community. Academic groups apply to be considered for the role, and the IMI decides which is best suited to partner with the industry participants. The funding is provided one-to-one by both the industry participant and the IMI. This approach is intended to scale funding and remove bottlenecks, giving industry undertakings direct access to academic expertise that would otherwise take years to materialize. Initiated in 2007, the IMI has had a budget of 5.2 billion euros, making it the largest public–private partnership in the world. It is therefore critical to review the successes of the initiative to date and determine areas for improvement as new projects are initiated for the future.

## IMI projects in support of neurological data science

A large portion of projects funded by the IMI, since its inception, have had a focus on neurodevelopmental, neurodegenerative or neuropsychiatric disorders, as these conditions collectively directly impact 1 in 3 people world-wide (2). As a leading cause of illness and of disability, these disorders significantly reduce human capabilities and productivity. By one estimate, the cost to the global economy is currently between $2.5 trillion USD and $8.5 USD trillion per year (3). Many IMI projects were focused on Alzheimer’s disease, where one might argue that, prior to 2008, little progress or investment had been made by industry, and only symptomatic treatments were available. At the genesis of the IMI efforts, we lacked precision medicine solutions for most of these and diseases and, even more discouragingly, many large drugmakers began to redeploy their investments away from neuroscience pipelines (4). This departure was hastened by stacked up clinical failures in the years prior, imminent patent expirations, and a lack of a pipeline caused by a combined lack of a biological or mechanistic understanding of these diseases and other challenges unique to brain research (e.g., blood–brain barrier) and exponentially rising costs of R&D.

Across many global initiatives (e.g., the European College of Neuropsychopharmacology (ECNP), the International College of Neuropsychopharmacology (CINP), the National Institutes of Health (NIH), etc.) common challenges were identified that were hampering progress, including, but not limited to:

- Defining the pathological phenotype precisely and ability to diagnose it.
- A lack of objective diagnostic tests and treatment responsive biomarkers.
- Intermediate phenotyping to characterize multiple risk factors and address pleiotropy of complex disorders.
- Mechanism-based modeling and simulation approaches for quantitative understanding of the pathology.
- New drug targets involved in the pathology of interest.
- Lack of translational validity: promising effects of novel compounds in animals did not reliably predict efficacious effects in patients.
- Novel tools and technologies for measuring the brain at a molecular, circuit, and systems level.
- Limitations of standard study designs (e.g., double-blind, randomized, controlled trials).
- Transfer of small molecules and biologics across the blood–brain barrier.

The field recognized that new large-scale, data collection and analysis efforts were necessary to understand these heterogeneous, polygenic disorders and would require broad collaboration and sharing of tools and data.

Significant re-investment and re-engagement by industry in neurodegenerative diseases, including AD, certainly correlates with IMI’s tenured investment. Generation of numerous databases and access to AD data, infrastructure for conducting large-scale trials, were fostered by IMI, as well as a new molecular-based taxonomy. It is indiscernible, however, whether these have been utilized or integrated into any of the drug programs (Aducanumab (Aduhelm^™^) and Leqembi (lecanemab-irmb)) that have recently been approved by the U.S. Food and Drug Administration (FDA). To evaluate the strengths and weaknesses of the IMI framework for advancing precision medicine solutions for neuroscience, we interviewed key contributors to six funded efforts: Aetionomy, the European Medical Information Framework (EMIF) project, the European prevention of Alzheimer’s dementia consortium (EPAD), eTRIKS, and Enhancing Quality in Preclinical Data (EQIPD) and Psychiatric Ratings using Intermediate Stratified Markers (PRISM), see Table 1. Total funding by IMI between IMI1 (2008–2013) and IMI2 (2014–2020) amounts to €5.276 billion. Of this €182,384,533 was invested in the initiatives outlined within this review (1).

**Table 1:** Overview of selected CNS and data management IMI initiatives.

IMI initiative	Dates	Project goals	Accomplishments	Resources
The AETIONOMY Project	01/01/2014 to 31/12/2018	Facilitate the use of precision medicine in neurodegenerative disorders	Integrated a broad range of datasets and dissected underlying mechanisms of disease; developed prototype Parkinson’s disease taxonomy	https://www.aetionomy.eu/
EMIF – The European Medical Information Framework Project	01/01/2013 to 30/06/2018	Improve access to patient-level data	Built a common information framework platform to streamline organization of and access to diverse data sources	http://www.emif.eu/
EPAD – The European Prevention of Alzheimer’s Dementia Consortium	01/01/2015 to 31/10/2020	Enhance design of clinical trials for AD drug candidates through adaptive trial design and focus on pre-symptomatic disease phase	Combined existing national and regional registers of potential pre-symptomatic AD patients and performed follow-up testing to characterize the subject pool	https://ep-ad.org/
The eTRIKS Collaboration	01/10/2012 to 30/09/2018	Centralize metadata for use in translational research	Developed a searchable IMI data repository and facilitated research collaboration	https://www.etriks.org/consortium/
EQIPD – Enhancing Quality In Preclinical Data	01/10/2017 to 30/09/2021	Improve the quality of data in non-regulated drug discovery research	Developed a novel quality management system and other open-access tools to help researchers generate reliable data	https://go-eqipd.org/
The PRISM Project	PRISM:01/04/2016 to 30/09/2019, PRISM 2:01/06/2021 to 31/05/2024	Develop a new understanding of neuropsychiatric disorders and an improved transdiagnostic approach	Conducted biomarker research across cohorts of patients with different conditions and overlapping symptoms to uncover common underlying pathologies	https://prism-project.eu/en/prism-study/

## IMI projects represented in this review

The EMIF project was a public–private consortium with 57 partners that operated for 5.5 years, from January 2013 to June 2018 with the goal of improving access to patient-level data. With patient data housed in disparate locations and in different systems, typically in isolation and not accessible from the outside, it is not possible to fully leverage its potential. EMIF sought to develop common technical and governance solutions and improve access and use of health data. To this end, EMIF built a common Information Framework (EMIF-Platform) to link up and facilitate access to diverse medical and research data sources. By integrating data from various sources such as electronic health records, biobanks, and clinical trials, EMIF enabled researchers to access a wealth of diverse and large-scale data sets. In its 5 years, EMIF successfully leveraged data from than 62 million EU adults and children through federal databases and cohorts from 7 different countries, improving access to and providing tools and workflows to discover, access, assess, and (re)use human health data. To explore whether the platform might be applicable across disciplines, EMIF included two therapeutic areas: AD (EMIF-AD, with a focus on pre-dementia AD) and metabolic complications of obesity (EMIF-Metabolic). Through this multi-disease approach, EMIF facilitated the identification of commonalities, patterns, and insights across diseases and patient populations, allowing researchers to gain a better understanding of disease mechanisms, risk factors, and treatment outcomes.

The EPAD consortium^2^ aimed to pioneer a novel, more flexible approach to clinical trials of AD drug candidates. To this end, the EPAD focuses on adaptive trial designs that will enable investigators to gather results faster and at a lower cost, with the pre-symptomatic phase of disease in mind for the prevention or delay of advanced symptom onset. Challenges to this approach include the difficulty of identifying people who are likely to develop AD, considering an inadequate understanding of early stages of the disease, as well as the lack of flexibility in how clinical trials are conducted. To overcome these challenges, EPAD pooled existing national and regional registers of individuals at risk of developing AD to create a single, pan-European register of around 24,000 people. Of these, the 6,000 deemed to be at greatest risk of AD were placed into a specialized, at-risk subject cohort that underwent standardized tests and follow-up. Finally, 1,500 of these subjects participated in early stage ‘adaptive’ clinical trials of therapeutics intended to prevent the progression of AD. The compiling and streamlining of these disparate datasets led to the identification of four distinct subgroups based on cognitive function (5).

The overall aim of the PRISM project^3^ was to develop a quantitative, transdiagnostic neurobiological approach to the understanding of neuropsychiatric disorders in order to accelerate the discovery and development of better treatments for patients with those disorders (6). Elucidation of common underlying pathologies across conditions could facilitate development of therapeutics that address those symptoms directly, outside of the constraints of treating the diseases as a whole. The development and implementation of such an innovative transdiagnostic framework requires a multi-staged approach. First, transdiagnostic and translational quantitative biomarkers need to be identified and implemented in clinical and pre-clinical domains. Second, proof-of-concept needs to be provided for identified biomarkers, showing that they allow for stratification of patients on the basis of quantitative biological measures. To this end, the project partners carried out a range of tests on patients with neuropsychiatric disorders (7) in a bid to determine which biological parameters can be matched with specific clinical symptoms like social dysfunction (8). They identified quantitative biological parameters that allowed the grouping of patients into clusters based on symptoms and underlying causes. For example, PRISM found that social dysfunction is transdiagnostically associated with default mode network disconnectivity in schizophrenia and Alzheimer’s disease (9). They also developed new behavioral readouts using passive remote smartphone monitoring with the aim of identifying novel digital biomarkers (10, 11). Finally, a preclinical testing battery with parameters homologous to those studied in patients was implemented to allow for back-translation of human findings and deliver predictive model systems to accelerate the drug discovery process (12). The PRISM project was one of the rare IMI endeavors that successfully received follow-on funding to build upon these results, through PRISM2 (13).

The Aetionomy project^4^ innovated classification approaches for neurodegenerative diseases by applying computational tools to molecular and biological data (mechanistic data) based data of Alzheimer’s disease (AD) and Parkinson’s disease (PD) that might contribute to a ‘taxonomy’ of these conditions, and help the community move towards a precision-medicine approach, instead of relying solely on clinical or symptom-based approaches. Aetionomy used a broad range of datasets, ranging from molecular to symptom data, and organized, structured, integrated them to dissect the underlying mechanistic causes in order to bring structure to the classifications. The consortium successfully demonstrated that their prototype taxonomy could be used to identify patient subgroups in Parkinson’s disease (PD). These efforts resulted in an open-access knowledge base with inventories of mechanistic hypotheses that form the basis for the prototype taxonomies.

The eTRIKS collaboration^5^ with ELIXIR-Luxemburg Node is an IMI data repository that centralizes ongoing and past IMI project level metadata for translational research scientists who require information about study projects. eTRIKS places an emphasis on the findability of research study descriptions with the aim of linking global data in a way that can be optimally leveraged to improve biomedical research, creating value for public and private organizations and driving research collaboration towards precision medicine. eTRIKS aimed to improve the technological platforms that scientists can use to share data.

The EQIPD project^6^ sought to generate simple and sustainable solutions to improve data quality in non-regulated drug discovery. One of the main outcomes of the project was a novel quality management system as well as a range of other open-access tools and learning materials. EQIPD sought to provide various stakeholders groups with resources that would facilitate collaboration and ensure generation of robust and reliable data. The EQIPD project, which was active from 2017 to 2021, was advanced by 30 consortium members as well as several dozens of stakeholders representing academic institutions, industry, CROs, academic core facilities, funders and research tool manufacturers. A non-profit organization Guarantors of EQIPD e.V. was founded in 2021 to maintain, further develop and to disseminate the project’s output.

## Lessons learned from a decade of the IMI

Drs. North and Haas conducted interviews and synthesized feedback from six of the program leaders, representing the academic and industry viewpoints and contributions to these IMI initiatives: Anton Bespalov, Hugh Marston, Martien Kas, Martin Hofmann-Apitius, Simon Lovestone, and Bart Vannieuwenhuyse. We asked each for their reflections on the strengths and weaknesses of the IMI approach (Table 2), with a focus on whether or not the unique governance and funding models worked as intended, whether the resulting platforms and data represented significant contributions to the field, and what pitfalls could be avoided in the future. The views in this section also represent those of the last author, who is not an EU citizen, who did not directly participate in any of these funded projects but, was integrally involved in all of them either at inception (generating & designing proposals, serving on a scientific advisory board, or serving as an independent reviewer on behalf of IMI) over a decade, and was invited to prepare this manuscript by the editors as an independent party.

**Table 2:** Pros and Cons to the IMI approach.

Pros and Cons to the IMI approach	
Positive impacts	Room for improvement
Correlated with major advancements in the field of AD research	Need for central management of databases and biomarker repositories
Unique, disruptive approach; emphasis on goal-oriented progress	No mechanism for sustainability of projects beyond funding period
Successfully fostered new ideas and transformed funding landscapes	Process for matching academic collaborators to industry sponsor not always successful; unclear alignment of IP and profit incentives
Facilitated new collaborations across industries/communities	Excessive bureaucratic and administrative burden

The fundamental question at hand is whether mechanisms like the one created by the IMI are indeed successful in advancing precision solutions for some of the over 800 diseases of the brain on behalf of EU citizens, or the world. First, one must ask, what are our metrics to assess such success? The number of new drug targets? Actionable advancement in disease understanding? Patents filed? Datasets generated? New investment by industry founded on the results within these programs?

Prior to the IMI, no comparable major public–private partnerships and investment had existed within this healthcare space. Thus, IMI was forced to blaze a trail that would scale across hundreds of projects and apply across geographies, addressing governance, program management, monitoring, and balancing of incentives between multiple interested parties. Many groups have since been able to leverage IMI protocols and procedures as templates to guide their own initiatives.

The overall impressions of the six contributors were aligned in that the IMI funding scheme provided a unique boost to collaboration unlike any other opportunity previously available. The authors felt that the initiative has been very successful, has fostered new thoughts and notions, and has even been transformative to both the EU and US funding and policy landscapes, noting that ambassadors from the IMI had spoken to a congressional panel to encourage more public–private partnership in the drafting of a landmark piece of US research legislation, the 21st Century Cures Act. One author summarized his perspective on the initiative as “totally thrilling, controversy-arising, and disruptive - in a good way.” Another author insisted that, compared to traditional funding approaches, “IMI is more impactful, has more traction in reality, and is more focused on solving problems.” Many authors noted that they felt they would not have accomplished what they had with a funding scheme other than IMI. Most importantly, the IMI has provided the unique opportunity for collaboration between a range of participants – industry, academia, and policymakers – where the expressed goal of each project is to meet the needs of patients. “When the goal is to improve patients’ lives, all stakeholders need to be involved from the beginning.”

The most widely cited advantage of the IMI approach was that it uniquely fostered collaboration between industry and academia early in a project’s life cycle and in a precompetitive “demilitarized zone.” The approach allowed partnerships that otherwise would have been competitive to instead be collaborative. Bringing academic partners into industry-driven initiatives is particularly important for facilitating multi-disciplinary work: it would not be possible for companies to employ experts in every relevant field of study pertaining to their project. The interviewees attested that “the IMI has boosted multi-disciplinary collaboration across Europe like never before.” Beyond the furtherance of collaboration *between* industry and academia, the IMI has also fostered collaboration *within* academia and *within* industry, as well.

Importantly, they observed that the initiatives and the “calls for funding” are industry-driven: the IMI allows industry to source academic support in a way that will accelerate industry initiatives, rather than industry attempting to piece together what they need from academic research that is already occurring. This allows the pharmaceutical partner to drive and explore proof of principle. Having industry input, as well as the input of other stakeholders such as patient groups, early in the research planning process is “crucial” to ensuring the studies are designed in a way that will benefit the drug development process. While academia produces important and interesting science, it is often not generated in form that is not workable for industry. One contributor even remarked that the “academic key opinion leaders have much less impact in [some of the IMI partnerships than they do] in other funding schemes, and this is a key to its success!”

While most contributors spoke about the advantages of IMI from an industry perspective, those who joined IMI projects from the academic side also cited numerous positive attributes. In general, IMI moved academic research forward, increased the amount of funding available for academic research, cross-academic fertilization, and collaboration across all of Europe in a way that has not happened in other cross-country funding schemes. Furthermore, the involvement of industry has encouraged a focused, goal-oriented ethos with elevated problem-solving capability and introduced industry-style project management, which is generally an improvement upon the administrative and operational capabilities of academic research centers. The projects have also led to a positive impact on flow of people between academic and industry. IMI has “taken the interesting science produced in academia and elevated it to a position of global impact.”

Despite the overwhelming positives the contributors recounted, IMI was described as having a number of disadvantages or areas for improvement. Most notably, the contributors felt that IMI projects were not sustainable and were in need of a mechanism for more follow-up time. The 3 year (extendable to 5 year) grant term was universally thought to be too short without a mechanism or framework for sustainability thereafter in place, and progress made in many of the projects was lost when the funding term came to an end. Several of the projects were proof-of-concept and need further investment and time to determine whether their results can be independently replicated (e.g., PRISM, AETIONOMY). Some of the projects had to establish independent sustainability models to ensure that the product of their efforts would be maintained after the funding cycle was over (e.g., EQIPD, eTRIKS). While some projects were selected for renewal, the majority were not. One author who did succeed in continuing funding of the project through a related grant noted that this had required perseverance and an innovative approach to the application process, but also a loss of valuable time. These cases suggest an opportunity for IMI to consider how it transitions projects at the end of their lifecycle to ensure that valuable (public) assets are not lost.

The need to generate digital data repositories and open-source software tools and promote data sharing were addressed by several of the programs. Indeed, eTRIKS was explicitly designed with the intention that all relevant IMI projects could utilize its common platform to avoid each project having to invest in their own knowledge management system and avoid duplicative efforts. However, there appears to be no central strategy within the IMI for knowledge management, central management of legacy data or establishment of common data standards across programs, although IMI-funded Neuronet^7^ seeks to address some of these challenges. The implementation of a strategy for an integrated, comprehensive international digital infrastructure for research data would be a substantive boon to the next generation of IMI. To realize such a transformative opportunity, we will need consensus and coordination across critical agencies, such as the European Medicines Agency (EMA), the U.S. National Institutes of Health (NIH) and the U.S. Food and Drug Administration (FDA), among others.

Similarly, the need for objective biomarkers, and the collection of biosamples across multiple centers, was a focus of several of the programs. However, each program addressed methodological issues of sample collection, handling, long-term storage, retrieval and analysis, independently, and under unique governance and practice frameworks. The establishment of an IMI core research infrastructure to promote high-quality, streamlined procedures under appropriate governance, leveraging best practice guidelines (14) for establishing repositories, is highly recommended.

The funding scheme employed by IMI in these initiatives is generally termed a “public–private partnership” (PPP). In a public–private partnership model, various stakeholders from the public and private sectors come together to collaborate on a shared goal or project. While such partnerships can yield numerous benefits, they also have the potential to give rise to conflicts of interest among the stakeholders involved. In a public–private partnership model involving academic and industry stakeholders, conflicts of interest can emerge due to the divergent goals and motivations of these two sectors. Academic stakeholders, such as universities and researchers, typically prioritize knowledge generation, particularly for discovery & innovation, academic freedom, and the pursuit of unbiased scientific inquiry and generation of publications. They strive to contribute to the advancement of knowledge and the public good. On the other hand, industry stakeholders, including corporations and businesses, may be driven by a focus on product development, reproducibility and robustness of results, and commercial interests, such as profit maximization, and gaining a competitive edge in the market. These differing priorities can lead to conflicts when it comes to issues such as research direction, data sharing, intellectual property rights, and publication of research findings. Academic stakeholders may seek to publish research that contributes to the public domain, while industry stakeholders may prefer to protect proprietary information. Additionally, concerns about potential bias or undue influence can arise when industry funding is involved in academic research, raising questions about the objectivity and integrity of the findings. Addressing these conflicts of interest requires clear guidelines, transparent communication, and robust safeguards to maintain scientific rigor, maintain academic independence, and ensure the public’s trust in the research outcomes.

Participants did, in fact, raise concerns about the process by which IMI “matches” academic applicants to the industry partners, where the inevitable mismatching of personalities or working styles of the groups, inherent differences in the academic and pharmaceutical working cultures, and unclear incentive alignment. While the industry participants indicated that they were able to provide guidance to the IMI to steer them towards the most suitable academic match, successfully influencing this process required significant finesse, diplomacy, and a bit of luck. In some cases, tension or disputes arose over the division of labor and assets: some industry participants bristled at the common desire among academics to work independently and without outside influence, and some academic partners wondered whether the industry partner was contributing enough to justify their ownership of the IP. Incentives (both for academics and for industry partners) within the IMI framework is a consistent area of challenge. One might consider whether new models can be developed that more equitably incentivize academic involvement in research that has the potential to lead to profits solely for private companies. The IMI could also consider whether the products generated by these programs are truly ‘translating’ into know-how, IP or technologies that can be readily incorporated into R&D efforts. Overall, while the mixing of backgrounds and IP incentives did lead to some real tension, the contributors felt that this was not all bad: the mingling of different viewpoints and goals, in their estimation, improved the perspective and understanding of all. Nevertheless, it is clear, that further investment to optimize the matching, incentives and IP and communication frameworks for PPP by IMI is required.

Finally, by strong consensus, one of the biggest detractors from the IMI experience was the overbearing administrative demands, although there were dedicated project management agencies engaged for much of the bureaucratic work. One author exclaimed that the red tape and bureaucracy were simply “crucifying.” There was a strong emphasis on formal reporting, for which deliverable timelines were highly important, while deliverable content was a mere formality. Some of the contributors felt that, in contrast to the IMI, the US NIH has a more practical approach to funding, with a stronger orientation towards goals and achieving results. Going forward, the IMI could accomplish more for drug development and better improve the lives of patients with more flexibility and a stronger emphasis on accomplishment, and less insistence on administrative procedures. Still, not all feedback in this area was negative: the application process was noted as being agreeable, with a reasonable amount of work required for the first round and the bulk of the application work only necessary for finalists who stood a high chance of success. Still, the benefits to science were unanimously seen as outweighing the bureaucratic frustrations. “IMI has had a lot of red tape, but for drug development, it has gotten it done.”

As the proverb goes, “hindsight is 20/20” and one can always find room for improvement. But if we look back at where neuroscience R&D was in 2007, before IMI was launched, and where we are headed now, with new tools and new insights largely driven by the trails blazed by IMI, one has to conclude that these were investments well worth making. Many of these efforts would now be categorized as ‘Learning Health Systems” models, seeking to achieve continuous rapid improvement in health and healthcare and to transform organizational practice. As such, perhaps future initiatives might be informed by the insights garnered in this field, including rapid evidence-to-implementation cycles and relevant metrics of success.

## Conclusion

The IMI approach to funding biomedical research has been a unique approach to fostering collaboration between industry and academia. Participants in a number of IMI projects shared their opinions that the funding scheme was highly successful and facilitated ideas and innovation that would not have occurred under other traditional funding mechanisms. The IMI approach could be strengthened through the addition of mechanisms to ensure sustainability of projects after initial funding terms, centralization of database and biomarker repository management, a better method of matching academic and industry partners, alignment of IP and profit incentives, and a reduction in bureaucratic administrative demands.

## Data availability statement

Publicly available datasets were analyzed in this study. This data can be found here: https://www.imi.europa.eu/projects-results/project-factsheets.

## Author contributions

HN and MH conducted interviews and drafted the manuscript. MH-A, MK, and HM contributed project-specific content and opinions for the review. All authors contributed to the article and approved the submitted version.

## Funding

The publication fees for this open-access manuscript were supported by Cohen Veterans Biosciences (New York, NY). Funding for Aetionomy [Grant #115568] EMIF [Grant #115372], EPAD [Grant #115736], eTRIKS [Grant #115446], EQIPD [Grant #777364], PRISM [Grant #115916], were all supported through the Innovative Medicines Initiative 1 & 2 Joint Undertakings.

## Conflict of interest

The authors declare that the research was conducted in the absence of any commercial or financial relationships that could be construed as a potential conflict of interest.

## Publisher’s note

All claims expressed in this article are solely those of the authors and do not necessarily represent those of their affiliated organizations, or those of the publisher, the editors and the reviewers. Any product that may be evaluated in this article, or claim that may be made by its manufacturer, is not guaranteed or endorsed by the publisher.

## Footnotes

^1^ http://www.emif.eu/

^2^ https://ep-ad.org/

^3^ https://prism-project.eu/en/prism-study/

^4^ https://www.aetionomy.eu/

^5^ https://www.etriks.org/consortium/

^6^ https://go-eqipd.org/

^7^ https://www.imi.europa.eu/projects-results/project-factsheets/neuronet

## References

[1] Available at: https://www.imi.europa.eu/about-imi/mission-objectives

[2] World Health Organization. Optimizing brain health across the life course: WHO position paper. WHO.Int, Available at: https://www.who.int/publications/i/item/9789240054561 (Accessed August 5, 2022)

[3] Trautmann S Rehm J Wittchen HU. The economic costs of mental disorders. EMBO Rep. (2016) 17:1245–9. doi: 10.15252/embr.201642951, PMID: 27491723 PMC5007565

[4] Wegener G Rujescu D. The current development of CNS drug research. Int J Neuropsychopharmacol. (2013) 16:1687–93. doi: 10.1017/S1461145713000345 23651558

[5] Ritchie CW Muniz-Terrera G Kivipelto M Solomon A Tom B Molinuevo JL. The European prevention of Alzheimer’s dementia (EPAD) longitudinal cohort study: baseline data release V500.0. J Prev Alzheimers Dis. (2020) 7:8–13. doi: 10.14283/jpad.2019.46, PMID: 32010920

[6] Kas MJ Penninx B Sommer B Serretti A Arango C Marston H. A quantitative approach to neuropsychiatry: the why and the how. Neurosci Biobehav Rev. (2019) 97:3–9. doi: 10.1016/j.neubiorev.2017.12.008, PMID: 29246661

[7] Bilderbeck AC Penninx BWJH Arango C van der Wee N Kahn R Winter-van Rossum I. Overview of the clinical implementation of a study exploring social withdrawal in patients with schizophrenia and Alzheimer’s disease. Neurosci Biobehav Rev. (2019) 97:87–93. doi: 10.1016/j.neubiorev.2018.06.019, PMID: 29940238

[8] Porcelli S Van Der Wee N van der Werff S Aghajani M Glennon JC van Heukelum S. Social brain, social dysfunction and social withdrawal. Neurosci Biobehav Rev. (2019) 97:10–33. doi: 10.1016/j.neubiorev.2018.09.012 30244163

[9] Saris IMJ Aghajani M Reus LM Visser PJ Pijnenburg Y van der Wee NJA. Social dysfunction is transdiagnostically associated with default mode network dysconnectivity in schizophrenia and Alzheimer’s disease. World J Biol Psychiatry. (2022) 23:264–77. doi: 10.1080/15622975.2021.1966714 34378488

[10] Jagesar RR Vorstman JA Kas MJ. Requirements and operational guidelines for secure and sustainable digital phenotyping: design and development study. J Med Internet Res. (2021) 23:e20996. doi: 10.2196/20996, PMID: 33825695 PMC8060862

[11] Jongs N Jagesar R van Haren NEM Penninx BWJH Reus L Visser PJ. A framework for assessing neuropsychiatric phenotypes by using smartphone-based location data. Transl Psychiatry. (2020) 10:211. doi: 10.1038/s41398-020-00893-4, PMID: 32612118 PMC7329884

[12] Peleh T Ike KGO Wams EJ Lebois EP Hengerer B. The reverse translation of a quantitative neuropsychiatric framework into preclinical studies: focus on social interaction and behavior. Neurosci Biobehav Rev. (2019) 97:96–111. doi: 10.1016/j.neubiorev.2018.07.018, PMID: 30660427

[13] Available at: https://www.imi.europa.eu/projects-results/project-factsheets/prism-2

[14] Campbell LD Betsou F Garcia DL Giri JG Pitt KE Pugh RS. Development of the ISBER best practices for repositories: collection, storage, retrieval, and distribution of biological materials for research. Biopreserv Biobanking. (2012) 10:232–3. doi: 10.1089/bio.2012.1025, PMID: 24844910

